# Clinical Considerations and Outcomes of Robotic Urologic Surgery in Obese Patients

**DOI:** 10.4274/TJAR.2023.231315

**Published:** 2024-05-03

**Authors:** Nazih Khater, Anna G. Morris, Delena M. Vanvalkenburg, Andrew J. Garcia, Kevin Jin, Shahab Ahmadzadeh, Sahar Shekoohi, Elyse M. Cornett, Alan David Kaye

**Affiliations:** 1 Department of Urology, Louisiana State University Health Sciences Center at Shreveport, Shreveport, Louisiana; 2 School of Medicine, Louisiana State University Health Sciences Center at Shreveport, Shreveport, Louisiana; 3 School of Medicine, Louisiana State University Health New Orleans, New Orleans, Louisiana; 4 Department of Anaesthesiology, Harbor-UCLA Medical Center, Torrance, California; 5 Department of Anaesthesiology, Louisiana State University Health Sciences Center Shreveport, Shreveport, Louisiana

**Keywords:** Anaesthesia, airway, complications, obese, urology surgery

## Abstract

Obesity is associated with many significant physiological changes. These considerations are important to surgery, especially in urological procedures. Obese patients often undergo surgical procedures and are at higher risk of complications. This investigation reviews physiological and anaesthesia considerations for obese and morbidly obese patients. In addition, urological surgeries and procedures should be considered for these higher risk patients. Clinical anaesthesiologists must use detailed assessment and, when appropriate, consultation in developing safe anaesthesia plans for these patients. Newer technologies have improved safety related to airway management, advanced airway devices, and regional anaesthesia with ultrasound-guided nerve blocks, which can reduce the need for opioids postoperatively. Recent developments in drug and monitoring technologies have also been developed and can be effective for obese and morbidly obese patients undergoing urological procedures and perioperative surgery, thus improving the likelihood of safety in this higher risk population.

**Main Points**

• Robotic pelvic surgery in obese patients presents with unique challenges.

• Specific ventilator settings and adjusting inspiration to expiration ratio, respiratory rate, and tidal volume, and utilizing pressure control ventilation can all help optimize respiratory function and prevent complications.

• Regarding cardiovascular Intraoperative strategies focus on maintaining adequate volume status and ensuring adequate mean arterial pressure (MAP).

• With respect to cardiovascular changes, intraoperative strategies focus on maintaining adequate volume status and ensuring adequate MAP

## Introduction

Robotic surgery is currently the most commonly adopted approach in minimally invasive urologic conditions. Obese patients are at a higher risk during surgical procedures, and robotic surgery may add more risk. Over the past 20 years, obesity in the United States (US) has increased tremendously. Based on the current available Centers for Disease Control and Prevention data, the prevalence of obesity in the US was 42.4% in 2017-2018. It appears that from 1999-2000 through 2017-2018, the prevalence increased from 30.5% to 42.4%. Concomitantly, the prevalence of severe obesity increased from 4.7% to 9.2%.^1^ The World Health Organization describes obesity based on body mass index (BMI) and includes 3 grades: Grade I (BMI between 30 and 34.9 kg m^2-1^), Grade II (BMI between 35 kg m^2-1^ and 39.9 kg m^2-1^), and Grade III (BMI of 40 kg m^2-1^ and higher). Grade III represents “morbid” obesity.^2^ Robotic surgery in obese patients represents a real challenge to the urologic surgeon and anaesthesiologist. Robotic pelvic surgery (prostate cancer, bladder cancer) puts the patient at risk for potential deep venous thrombosis (DVT), pulmonary embolus (PE), and increased intracranial pressure (ICP). Obesity adds more complexity to the surgical procedure, resulting in prolonged operative time related to challenges in patient positioning, excessive intra-abdominal fat, delicate surgical planes, and sometimes pelvic lipomatosis that may narrow the robotic surgical field of view.^3,4^ Morbid obesity may prolong the operative time due to difficulty reaching the pelvis with the robotic arms, in addition to special considerations with trocar selection.^5^ Prolonged operative time would expose the patient to more hemodynamic changes and anaesthetic considerations. Robotic kidney surgery (for kidney cancer and other benign renal conditions) has also been studied by Kott et al.^6^ where an increased BMI above 30 kg m^2-1^ has been shown to contribute to post-operative complications (POC) in patients undergoing robotic assisted partial nephrectomy. Therefore, the present investigation aimed to analyze the most current anaesthetic and surgical considerations of robotic urologic surgery in morbidly obese patients. We analyzed a recent literature to attempt to recognize any common findings regarding patient BMI and surgical outcomes and/or complications.

### Methodology

A literature review was performed on previously published manuscripts on robotic urologic surgery in obese patients. This included evaluation, anaesthetic considerations, and expected hemodynamic changes that may occur. The databases searched in this investigation included PubMed and Google Scholar, and 53 published manuscripts were reviewed.

## Clinical and Research Consequences

### Current available robotic surgeries in urology

Robotic surgery in urology was introduced in the early 2000s. Robotic prostatectomy, more commonly known as robotic-assisted laparoscopic prostatectomy (RALP), was performed in the US in 2000. Since then, almost every open urologic surgery has been performed robotically. Robotic surgery platforms have also evolved from the multi-port DaVinci S, Si, and Xi to the most recent single-port SP platform. Robotic surgery is currently available for prostate, kidney, bladder, and other urologic pathologies (Table 1).

### Hemodynamic changes that occur during robotic surgery in obese individuals compared with non-obese individuals

Robotic assisted surgery offers many potential benefits for obese patients, including reduced fasciotomy size, decreased postoperative pain, and reduced postoperative wound complications, which are more prevalent in obese patients than in non-obese patients.^7^ However, this type of surgery also causes physiological stresses on the body that can be even more dramatic in obese patients. Obesity significantly alters the physiology of different organ systems, including the cardiovascular and pulmonary systems. These complex changes are especially relevant considerations when planning and performing robotic surgery because the physiologic burden caused by the surgery can cause further decompensation in this patient population.^8^ At baseline, obese patients have increased cardiac work because of increases in stroke volume and cardiac output (CO) and decreases in vascular resistance. These changes lead to hypertension and ventricular hypertrophy, which can eventually lead to congestive heart failure. These changes also cause increased pulmonary artery pressure and worsen cardiac dysfunction. Obesity causes changes in pulmonary physiology that follow a restrictive pattern. Elevated intra-abdominal pressure due to excess weight can reduce lung and chest wall compliance. It also causes lower functional residual capacity (FRC) and expiratory reserve volumes (ERVs), which can cause rapid oxygen desaturation and increase the likelihood of atelectasis.^9^

Abdominal insufflation is required to facilitate the movement of surgical instruments; however, the resultant pneumoperitoneum significantly alters hemodynamic properties.^7^ Pneumoperitoneum increases heart rate, systemic vascular resistance, and mean arterial pressure (MAP). This is likely due to compression of the major abdominal vessels, and smaller mesenteric vessels may also show changes in flow. Compression of vessels can also increase the likelihood of venous stasis and thromboembolism, for which obesity is also an independent risk factor. Increased baseline intra-abdominal pressure in the obese population can exacerbate these changes. Pneumoperitoneum also decreases respiratory function due to cranial displacement of the diaphragm, decreased FRC and ERV, and increased atelectasis and airway resistance, all of which are already present in obese individuals.^10,11^ Other complications can arise because the carbon dioxide used to insufflate the abdomen can be absorbed, leading to increased partial pressure of carbon dioxide. In non-obese patients without comorbid lung disease, this increase can be normalized by increasing minute ventilation and decreasing intraperitoneal CO_2_ pressure to prevent hypercapnia. However, lung pathology caused by obesity can prevent these compensatory mechanisms, leading to respiratory acidosis and hypercapnia.^7,12^ Hypercapnia can cause cardiac arrhythmias, pulmonary vasoconstriction, and autonomic nervous system stimulation, resulting in tachycardia and increased cardiac contractility. However, concomitant acidosis can depress myocardial contractility.^13^

Steep Trendelenburg positioning (STP) provides favorable surgical exposure in lower abdominal and pelvic robotic surgeries and is therefore strongly recommended. However, this position causes significant haemodynamic changes and is especially unfavorable for obese patients.^9^ This position further increases the compression of the diaphragm already present at baseline in obese individuals and is increased by intra-abdominal insufflation. Therefore, this position further increases the volume of atelectasis and the risk of hypoxemia.^7^ Additionally, due to the 30-40 degree angle at which patients are at during this positioning, significant decreases in both stroke volume and CO can be expected.^14^ Additional risks for STP include nerve injury, especially in the brachial plexus due to stretch injury, excess pressure on the head may lead to cervical spine injury, and prolonged lithotomy positioning increases patients’ risk for compartment syndrome, rhabdomyolysis, and increased intraocular and/or ICP^9,15^.

## Risks of Robotic Pelvic Surgery in Obese Patients

### Risks for obese patients undergoing robotic pelvic surgery

An individual is defined as obese when the BMI is >30 kg m^2-1^ and morbidly obese when BMI is >35 kg m^2-1^.^5^ For obese patients, robotic pelvic surgery poses several risks that involve physiological aspects of the cardiovascular and pulmonary systems, central nervous system, and special positioning of the patient.

Risks related to the cardiovascular system include decreased mesenteric blood flow, elevated MAP, elevated central venous pressure (CVP), decreased CO, and greater blood loss^11^. RAL procedures require STP, a method in which the patient’s head is slightly angled down, and abdominal insufflation with carbon dioxide to adequately visualize the abdominal contents adequately^9^. These two techniques increase intrabdominal pressure and compress abdominal arteries, leading to decreased mesenteric flow, increased systemic vascular resistance, and increased MAP, which can result in small decreases in CO.^9^ When combined with pneumoperitoneum, which adds cephalad pressure by abdominal contents pressing on the diaphragm, STP increases intrathoracic pressure, leading to increased CVP.^9^ Lindner et al.^16^ observed greater blood loss in obese patients than in non-obese patients during open radical retropubic prostatectomy.

Risks related to the pulmonary system include hypercapnia, atelectasis, hypoxia, hypoxemia, hypoventilation, apnea, respiratory distress, decreased lung compliance (LC), and reduced FRC and ERV.^9^ Hypercapnia can develop from the use of pneumoperitoneum with carbon dioxide via the absorption of gas from the insufflated abdomen,^9^ especially in the presence of a ventilation/perfusion (V/Q) mismatch or underlying lung pathology, such as chronic obstructive pulmonary disease, which would inhibit adequate compensatory changes.^9^ Additionally, because increasing evidence supports lower tidal volumes for ventilation in obese patients, a higher fraction of inspired air may be needed to maintain oxygenation, therefore increasing the risk of atelectasis leading to hypoxia and further exacerbating hypercapnia.^9^ pneumoperitoneum can cause atelectasis, which increases the volume of atelectasis in dependent lung regions.^11^ Obese patients are at increased risk for hypoxemia and may demonstrate arterial oxygen insufficiency compared with non-obese patients, which can be due to increased venous admixture and pulmonary shunt, as seen by the increased alveolar to arterial oxygen gradient of partial pressure of oxygen.^17^ Trendelenburg positioning may exacerbate this effect.^18^

In addition, obese patients commonly suffer from obstructive sleep apnea (OSA) and obesity hypoventilation syndrome (OHS), which greatly increase the risk of pulmonary complications in the postoperative period. Even mild cases of OSA threaten serious complications when combined with narcotics and general anaesthesia.^4,19^ Grieco et al.^18^ warn that intraoperative pressure control ventilation (PCV) could lead to severe alveolar hypoventilation in patients with airway opening pressures greater than 15 cm H_2_O. Wiltz et al.^3^ reported a significantly higher incidence of aborted procedures in obese patients due to respiratory distress. A risk of decreased LC may be observed with pneumoperitoneum and prolonged Trendelenburg positioning at a 40-degree to 45-degree angle in extremely obese patients.^10,20^ Decreased FRC and ERV can have both intraoperative and postoperative consequences. If the FRC is depressed below the closing capacity, patients can experience airway closure and subsequent hypoxemia, leading to rapid desaturation with hypoventilation, apnea, or respiratory failure. These effects are amplified by STP, pneumoperitoneum, and general anaesthesia.^9,10^ Risks involving the airway include subcutaneous emphysema from pneumoperitoneum, airway edema from fluid administration and prolonged Trendelenburg positioning, respiratory depression, airway closure, desaturation events, need for reintubation, and aspiration.^4,9,20^ Patients with a history of OSA or OHS are at increased risk of respiratory depression both intraoperatively and postoperatively related to commonly used intraoperative agents, including sedatives, neuromuscular blockade agents, analgesics, and residual anaesthesia.^9^ The risk of airway closure is increased in patients with STP.^17^ Grieco et al.^18^ reported severe expiratory flow limitation and airway closure in 22% of patients after Trendelenburg positioning. Postoperatively, obese patients are at increased risk of reintubation and desaturation events, especially those with OSA.^9^ An increased risk of aspiration may occur due to increased respiratory workload, higher gastric residual volumes, and increased difficulty of intubation in obese patients.^4,21^

Risks related to the central nervous system include decreased cerebral and ocular perfusion pressure, increased intracerebral and intraocular pressure (IOP), and ischemic optic neuropathy leading to vision changes or vision loss.^9^ Cerebral perfusion pressure initially increases with STP; however, it may decrease throughout the procedure due to head-down positioning and rising CVP. In contrast, intracerebral pressure (ICP) increases because of hypercarbia, causing cerebral vasodilation, increased intraperitoneal pressure, and increased intrathoracic pressure. As a result, CSF drainage is decreased and ICP subsequently increases. STP can lead to ischemic optic neuropathy due to elevated IOP and decreased ocular perfusion pressure. Obesity can further exacerbate CVP and end-tidal carbon dioxide elevations and lead to longer surgical duration, causing further increases in IOP^9^. Risks related to special positioning include worsening of obesity-related respiratory disorders, brachial plexus nerve injury, rhabdomyolysis, compartment syndrome, eye injury, cervical spine injury, dermal injury, and robotic trocar site-related injuries.^1,2,18^

Obese patients may be more sensitive to special positioning commonly used in robotic pelvic surgery, namely STP and pneumoperitoneum.^4^ Grieco et al.^18^ reports that in bariatric laparoscopic surgery, pneumoperitoneum and Trendelenburg positioning worsen obesity-related respiratory disorders and increase the anaesthetic risk. STP may cause a brachial plexus injury due to stretching between the shoulder and neck. Obese patients are especially at risk for mechanical cephalad slippage on the table due to STP and therefore require the use of various braces and pads, which can further injure the brachial plexus, especially with the use of shoulder braces and beanbag positioners.^9^ Because of traumatic compression of muscle tissue during extended surgical procedures, extremely obese patients are at increased risk for postoperative rhabdomyolysis because of their excessive weight, which can induce hypocalcemia.^2,5^ One study found that rhabdomyolysis, compartment syndrome, peripheral nerve injuries, and eye injuries were the most frequent positioning complications in patients with robotic-assisted radical prostatectomy (RARP).^22^ Prolonged lithotomy positioning similarly increases the risk of several perioperative complications, including common peroneal nerve injury, compartment syndrome, and rhabdomyolysis.^23^ The risk of eye injuries includes corneal foreign bodies, visual disturbances, and vision loss in one eye.^22^ Cervical spine injury risk is increased with the use of devices that place excess pressure on the head, and the risk of slippage of the patient increases with increasing weight.^9^ Should supportive positional devices fail and the patient slip while the robotic system is engaged, they are at increased risk of dermal, nerve, and incisional tears at robotic trocar sites.^9^ Acquisition of pressure injuries, of which obese patients are at great risk, can lead to further skin breakdown.^24^ In a non-experimental study that identified risk factors for pressure injury in surgical positioning, Menezes et al.^22^ found a significant association between patients with a BMI 30 kg m^2-1^ and risk of pressure injuries (Table 2).

### Recent Studies

Recent studies have shown additional risks of robotic pelvic surgery in obese patients. A summary of these results is presented in Tables 3 and 4.

A 2020 retrospective cohort study by Kott et al.^6^ investigated the association between obesity and the rate of POC following RALP nephrectomy (RPNx). The study revealed an association between BMI and POC in patients undergoing RPNx. The rate of POC was found to be higher in patients with a BMI above the inflection point (30 kg m^2-1^) and lower with increasing BMI up until the inflection point. Paradoxically, these results showed that overweight and mildly obese patients have a lower risk of POC after RPNx, and both morbidly obese and underweight patients have the most significant risk of developing POC. Overall, these data suggest that BMI may be an essential factor for clinicians and surgeons to consider in managing patients undergoing RPNx^6^.

A 2019 retrospective cohort study by Knipper et al.^25^ investigated the effects of obesity on perioperative and various early postoperative complications with stratification based on surgical approach, robot-assisted vs open radical (RARP vs ORP) in patients undergoing RP. The study found that for both RARP and ORP, obese patients had higher overall perioperative complications, total hospital costs, and longer length of stay compared with non-obese patients, as well as more cardiac, respiratory, and genitourinary complications following RP. In addition, although RARP was associated with higher total hospital costs, it had a more favorable complication profile than ORP. Overall, these data suggest that obesity is a significant risk factor for perioperative complications during RP and that RARP may be more beneficial than ORP in preventing adverse outcomes.^25^

A 2020 retrospective analysis by Han et al.^26^ investigated the effects of obesity on perioperative outcomes, including blood transfusion rates, intraoperative and postoperative outcomes, total costs, and healthcare resource utilization following RA laparoscopic RP (RALRP).^26^ The study found that patients diagnosed with class I-II obesity (BMI 35-39.99 kg m^2-1^) and morbid obesity (BMI ≥40 kg m^2-1^) experienced greater overall postoperative complications than non-obese patients. Additionally, morbidly obese patients experienced more adverse perioperative events, including overall, cardiac, respiratory, and genitourinary complications, increased hospital length of stay, and 12% higher costs. Overall, these data suggest that in patients undergoing RALRP, morbid obesity is associated with poor perioperative outcomes, requiring close management by physicians both in and out of the operating room.^26^

A 2020 multicenter retrospective cohort study by Nik‐Ahd et al.^27^ investigated the association between obesity and positive surgical margins (PSMs) in patients undergoing RALP versus retropubic RP (RRP). The study found that at all locations except the bladder neck, higher BMI was associated with increased odds of overall, peripheral, and apical PSMs among all patients. In addition, there was a significant association between obesity and peripheral PSMs in men undergoing RRP, but not RALP. Higher BMI is a risk factor for PSMs, and the association between obesity and PSMs is slightly stronger for men undergoing RRP than for those undergoing RALP.^27^

A 2018 meta-analysis by Wei et al.^28^ investigated the effects of obesity on long-term urinary incontinence (UI) following robotic-assisted laparoscopic RP (RLRP). The study found a significant association between obesity and UI in patients undergoing RLRP. When the surgical methods were stratified into laparoscopic RP and RLRP, the results indicated that obesity increased UI risk in patients who underwent RLRP but not LRP at 24 months. Therefore, obesity is associated with an increased risk of UI in patients undergoing RLRP at 12 and 24 months.^28^

A 2019 retrospective observational analysis by Yu et al.^29^ investigated the incidence and risk factors of postoperative pulmonary complications (PPCs) in patients who underwent RALP under specific conditions, including pneumoperitoneum and STP. The study found that in addition to other risk factors such as age >65 years, hypoalbuminemia, and inadequate positive end-expiratory pressure, higher BMI was associated with an increased risk of PPCs in patients undergoing RALP. In addition, prostate cancer patients with PPCs experienced more admissions to the intensive care unit (ICU), longer ICU length of stay, higher hospital costs, and higher overall morbidity and mortality. Overall, these data suggest that obesity could potentially lead to PPCs in patients undergoing RALP, which may increase the difficulty of care management for both the anaesthesiologist and surgeon and lead to higher rates of ICU admission, length of stay, and overall morbidity and mortality.^29^ A 2018 retrospective analysis by Porcaro et al.^30^ investigated the effects of clinical factors such as BMI on the risk of grade 3 Clavien-Dindo complications (CDCs) in patients having RARP with extensive pelvic lymph node dissection (EPLND). Clavien-Dindo grade 3 complications are defined as moderate-to-severe complications leading to lasting disability or organ resection and requiring surgical, endoscopic, or radiologic intervention, with or without the addition of general anaesthesia.^31^ The study found that increasing BMI was an independent predictor of a higher rate of grade 3 CDCs, which increased by 18.4% for each unit increase in BMI. Specifically, possible complications after RARP included ureteral injury, anastomotic urinary leakage, and symptomatic lymphocele. Overall, these data suggest that BMI is a risk factor for grade 3 CDCs in patients undergoing RARP with EPLND.^30^

A 2021 double-blind, placebo-controlled study by Pathak et al.^32^ investigated the effects of obesity on quality indicators such as length of stay and readmission after minimally invasive-radical retropubic prostatectomy (MI-RRP). The study found that obese patients experienced prolonged length of stay, increased readmission rates, and higher overall morbidity than non-obese patients, and there was a negative correlation between obesity severity and quality indicators. Overall, these data suggest that in patients undergoing MI-RRP, obesity increases the risk of poor quality outcomes; therefore, physicians should carefully consider these risks and discuss them with their at-risk patients.^32^

## Discussion

### Reducing Complications in Obese Patients Undergoing Robotic Surgery

Robotic pelvic surgery in obese patients presents unique challenges. From an anaesthesia standpoint, accounting for both the physiological changes associated with obesity and the impact of patient positioning is paramount.

With respect to cardiovascular changes, obese patients may present with elevated CVP and decreased mesenteric blood flow during robotic surgery, particularly in the STP^33^. Intraoperative strategies focus on maintaining adequate volume and MAP levels. Placing an arterial line can help monitor unpredictable hemodynamic responses such as hypertension or hypotension, bradycardia, and tachycardia.^34^ At the conclusion of surgery, when the legs are lowered from lithotomy, blood pressure should be measured to ensure that patients can tolerate the translocation of blood volume from the central compartment back into the lower extremities.^34^

With respect to respiratory changes, increased intra-thoracic pressure, decreased lung and chest wall compliance, and pneumoperitoneum can lead to decreased FRC, decreased ERV, hypoxia, hypercapnia, and atelectasis.^35^ Specific ventilator settings, including higher positive end expiratory pressure, maintaining peak inspiratory pressure (PIP) <40 mmHg, adjusting inspiration to expiration ratio, respiratory rate, and tidal volume, and using PCV, can help optimize respiratory function and prevent complications.^34,36^ In addition, patients who are obese or have OSA should be extubated while awake to prevent complications during extubation.^8^

With regard to airway optimization, increased adipose tissue in the face, neck, and abdomen can complicate patient positioning, neck extension, bag-mask ventilation, and tracheal intubation.^37^ The placement of the endotracheal tube helps prevent airway collapse and pulmonary aspiration via positive pressure ventilation to decrease atelectasis and V/Q mismatch. Video laryngoscopy or transnasal humidified rapid insufflation ventilation exchange can provide an early definitive airway in obese patients. Rechecking endotracheal tube positioning once in the STP or lithotomy positions to ensure that the tube does not displace into the mainstem bronchus is imperative.

Concerning the CNS, ICP and IOP due to increased cerebral blood flow and venous congestion may occur during Trendelenburg positioning and pneumoperitoneal initiation.^34^ While the STP position leads to an increase in IOP comparable to patients with glaucoma who have discontinued medication, no increased incidence of ischemic optic neuropathy has been observed in this setting. Addressing CNS risks includes achieving adequate MAP to maintain cerebral oxygenation in addition to preoperative consultation with an ophthalmologist for patients at risk of increased IOP.

**Figure 1:**
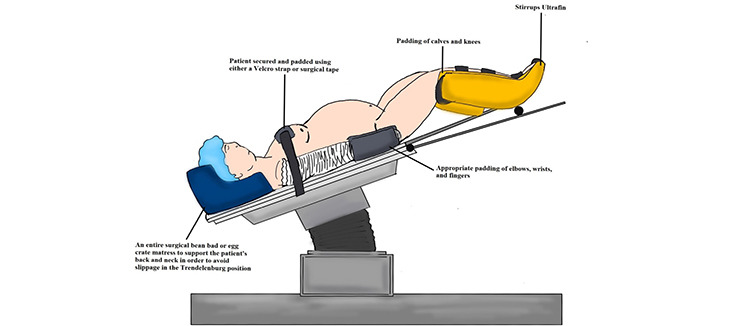
Trendelenburg positioning in the obese patient (drawing from Dr. Zoey Harris, MD, with permission)

Regarding patient positioning, going into the STP position is difficult in obese patients because of the excessive weight and skin laxity that can risk displacement on the operating room table. Patient positioning may be optimized using egg crates, vacuum-molded bean bags, shoulder supports, memory foam mattresses, gel pads, and OR table extenders.^8^ Other pertinent measures include having extra OR personnel assist with moving obese patients at the beginning and end of surgery.^38,39,40,41^ Figure 1 illustrates appropriate Trendelenburg positioning for obese patients.

### Recent Studies

Recent studies have shown how conditions can be optimized for robotic or laparoscopic pelvic surgery in obese patients. These results are listed in Tables 5 and 6. A 2020 retrospective analysis by Wilson et al.^39^ investigated the effects of a weight loss program before RARP in obese men with prostate cancer. The study found that after a median of 29 days on the weight loss program, patients presented with significantly reduced weight, percent body fat, and overall fat mass, all associated with less surgery-related adverse effects. These data suggest that undertaking a weight loss program in preparation for robotic pelvic surgery may benefit postoperative outcomes.^39^

A 2018 prospective non-randomized study by Blecha et al.^40^ investigated the impact of obesity on pulmonary deterioration in patients undergoing RARP. The study found that BMI was a significant predictor of increased PIP, peak driving pressure (P_drive_), and decreased LC, further exacerbated by STP and capnoperitoneum. So, BMI can be used to predict changes in PIP, P_drive_, and LC in the pre-operative setting.^40^

A 2019 double-blind randomized study by Gad et al.^41^ investigated the effects of PCV with volume-guaranteed (PVC-VG) versus volume-controlled ventilation (VCV) with equal ratio ventilation in obese patients undergoing laparoscopic hysterectomy. The study found that PCV-VG led to significantly lower PIP values and higher dynamic compliance than VCV. In summary, PCV-VG is superior to VCV in obese patients undergoing laparoscopic surgery in the Trendelenburg position.^41^

A 2018 observational study by Jun et al.^42^ investigated the effects of mannitol on optic nerve sheath diameter (ONSD) as a surrogate for ICP during RARP. The study found that ONSD was decreased in STP at 3 time points up to 90 min after the initiation of mannitol Mannitol administration may provide a valuable preventive measure to patients at risk of increased ICP during RARP, including obese patients.^42^

## Conclusion

Robotic urologic surgery in morbidly obese patients is very challenging but achievable. A multi-disciplinary team approach is primordial, and a dedicated anaesthesia team would lower the morbidity risk, allowing patients to undergo their planned procedure. Some pre-surgical risk factors to consider include hemodynamic changes such as hypertension and ventricular hypertrophy that can cause pulmonary hypertension and pulmonary changes such as reduced lung and chest wall compliance due to increased intra-abdominal pressure from excess weight. These factors can be exacerbated by many components, such as abdominal insufflation leading to pneumoperitoneum, STP, and use of carbon dioxide leading to complications such as DVTs due to vein compression, risk of hypoxemia or hypoxia, and hypercapnia. These potential complications lead to increased anaesthesia risks.

Studies have shown a positive correlation between patient obesity and blood loss during pelvic surgeries. Likewise, a significant increase in the number of aborted procedures related to respiratory complications has been identified in obese patients, citing expiratory flow limitation and airway closure as the chief reasons. Other correlations include increased risk of nerve damage, postoperative rhabdomyolysis, and hypocalcemia in patients with higher BMI. Other studies have identified a BMI of 30 kg m^2-1^ as significantly associated with increases in POC mentioned above, and even with increased odds of PSMs and Clavien-Dindo grade 3 complications.

While more data helps solidify this correlation, the risks of the complications listed are correlated with a BMI of 30 kg m^2-1^ and are even more significant as BMI increases. However, there are steps that physicians and surgeons can take to reduce these risks, such as placing arterial lines to monitor hemodynamic changes, adhering to specific ventilators, and extubation to minimize pulmonary risks. In addition, the use of egg crates or vacuum-molded bean bags to reduce the risks associated with STP to ensure that the patient can proceed with the procedure as planned.

## Figures and Tables

**Table 1:**
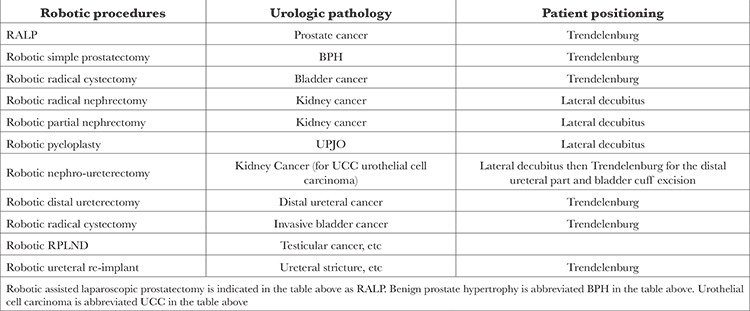
Most Performed Robotic Urologic Procedures (Using the Multi-port DaVinci Xi Platform)

**Table 2:**
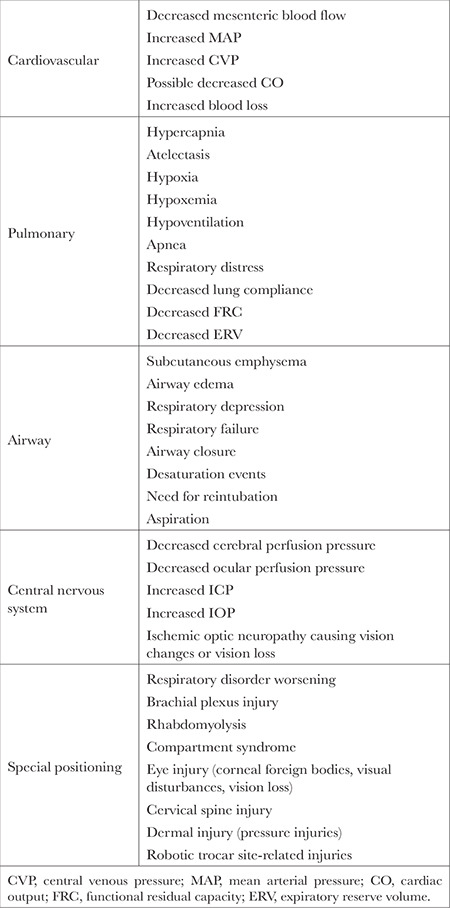
Risks Associated with Obesity in Robotic Pelvic Surgery

**Table 3:**
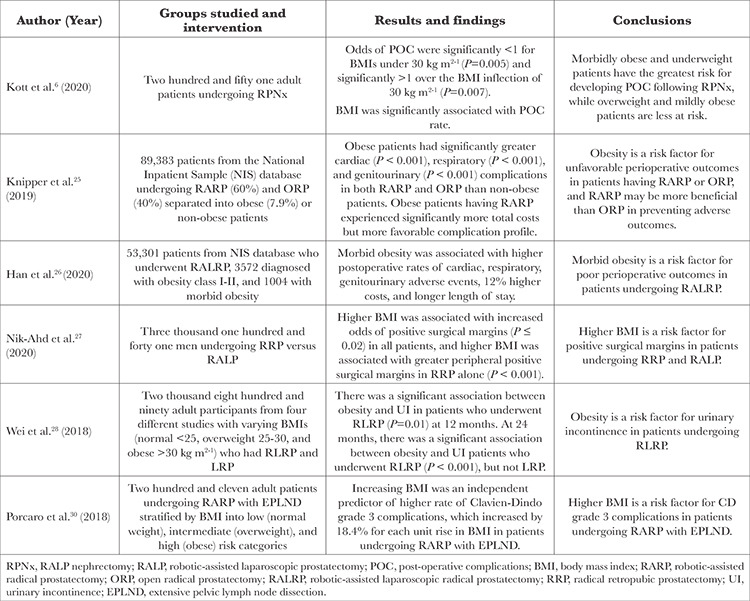
Clinical Efficacy and Safety

**Table 4:**
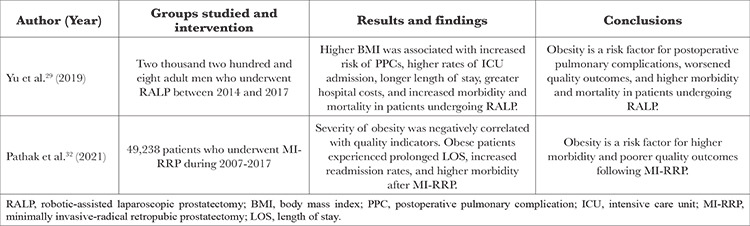
Comparative Studies

**Table 5:**
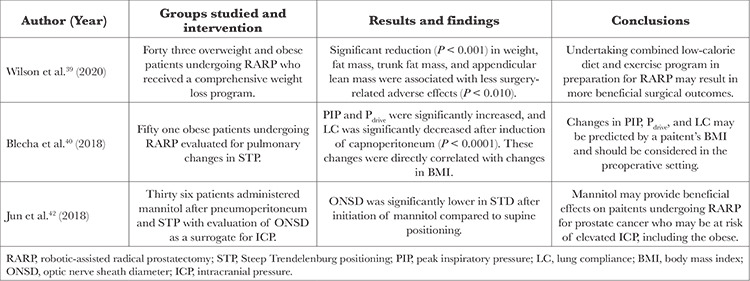
Clinical Efficacy and Safety

**Table 6:**
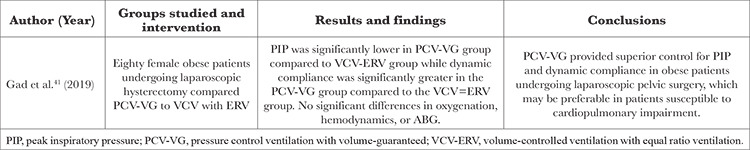
Comparative Studies
